# Apixaban Prophylactic Anticoagulation in Patients with Nephrotic Syndrome

**DOI:** 10.1055/a-1920-6224

**Published:** 2022-10-07

**Authors:** Tess Van Meerhaeghe, Alexandre Cez, Karine Dahan, Emmanuel Esteve, Ismail Elalamy, Jean Jacques Boffa, Eleonore Ponlot

**Affiliations:** 1 Department of Nephrology and Dialysis, Hôpital Erasme ULB, Brussels, Belgium; 2 Department of Nephrology and Dialysis, Assistance Publique Hôpitaux de Paris, Hôpital Tenon, Paris, France; 3 Hematology and Thrombosis Center, Hôpital Tenon, Hôpitaux Universitaires de l'Est Parisien, Assistance Publique Hôpitaux de Paris, Faculté de Médecine, Sorbonne Université, Paris, France; 4 Research Group Cancer, Haemostasis and Angiogenesis, INSERM U938, Centre de Recherche Saint-Antoine, Institut Universitaire de Cancérologie, Faculty of Medicine, Sorbonne University, Paris, France; 5 Sorbonne Université, INSERM UMRS1155, Hôpital Tenon, Paris, France; 6 Department of Obstetrics and Gynaecology, The First I.M. Sechenov Moscow State Medical University, Moscow, Russia; 7 Departement of Nephrology and Dialysis, Grand Hôpital de Charleroi GHDC, Charleroi, Belgium

**Keywords:** nephrotic syndrome, anticoagulation, direct oral anticoagulants, thromboembolic event, thromboprophylaxis, hypoalbuminemia

## Abstract

**Background** Nephrotic syndrome (NS) is associated with an increased risk of thromboembolic events (TEs), due to hemostatic derangements. The use of direct oral anticoagulants (DOACs) in the prevention of TE has not been studied intensively in patients suffering from NS.

**Methods** The method included retrospective analysis of consecutive incident patients with NS due to glomerular disease, receiving apixaban for thromboprophylaxis. It is an uncontrolled, single-center study.

**Results** We identified 27 patients treated with apixaban for the prevention of TEs, in the context of NS. During follow-up, apixaban minimal blood concentration (trough level; Cmin) and maximum blood concentration (Cmax) levels were measured. The mean duration of the anticoagulant treatment was 153 days (±132). Patients were followed for a mean of 14.7 months (±8.4) since the introduction of apixaban. Three patients had a TE at the time of NS diagnosis. Two patients had pulmonary embolism (PE) and one patient presented a stroke in a lupus membranous nephropathy context. One patient developed PE approximately 2 months after the introduction of apixaban treatment. No minor or major bleeding events were noticed.

**Conclusion** The present study shows that patients, suffering from severe NS under anticoagulant therapy with apixaban had a reduced risk of venous and arterial TEs compared with patients previously described in the literature, without increased risk of bleeding.

## Introduction

Nephrotic syndrome (NS) is characterized by heavy proteinuria and hypoalbuminemia. Patients with NS are known to have a prothrombotic state with an increased risk of thromboembolic events (TEs). The hemostatic derangements are not completely understood, but urinary losses of anticoagulant proteins may play an important role. 1 The estimated prevalence of thromboembolic disease can increase up to 37% in this specific population. 1

Moreover, the risk of thromboembolism may be influenced by the type of glomerular disease (membranous nephropathy [MN] being at higher risk, estimated at 37%), the duration of the NS, and the severity evaluated by serum albumin and proteinuria levels. 2 3 4 The majority of venous TE occurs within the first 6 months after NS diagnosis. 5

Some studies support the use of a prolonged thromboprophylaxis strategy in NS patients. 6 7 8 While classically warfarin has been used as an anticoagulant in NS, direct oral anticoagulants (DOACs), such as apixaban, are increasingly used to treat arterial or venous thrombosis in the general population. However, the efficacy and safety of DOACs in patients with NS have not been evaluated in large randomized controlled trials. Evidence is mostly based on case reports and case series. 9 10 11 12 13

Routine therapeutic monitoring of plasma anti-Xa activity is not required because of the drug's predictability and its wide therapeutic index. Despite the use of fixed doses of apixaban, determination of the amount of drug present in a given individual may be of interest in some vulnerable and unstable clinical situations, such as preoperative settings, bleeding episodes, complex comorbidities, extreme body weight, or elderly patients. 14 Hypoalbuminemia itself can lead to altered pharmacokinetics and pharmacodynamics of medications, although this topic is infrequently considered in daily clinical practice. 15

The aims of our study were to evaluate the efficacy of apixaban to prevent TE in patients with NS and to determine the interest of drug monitoring in these frail patients.

## Materials and Methods

### Design

We conducted a retrospective analysis of consecutive incident patients with NS due to glomerular disease, receiving apixaban for thromboprophylaxis. It is an uncontrolled, single-center study. The patients were included from September 2018 to 0ctober 2020. All patients consented to participate in the study.

NS was defined by the presence of urinary protein excretion greater than 3 g/day or as a protein/creatinine ratio of >3 g/g on urinary spot in association with hypoalbuminemia <30 g/L.

Patients who were already treated with anticoagulants at NS onset for another indication or with formal contraindication for anticoagulants were excluded.

All TEs were recorded at the onset of NS and during follow-up, as well as minor and major bleeding events.

### Intervention

Patients were systematically treated with apixaban when albumin levels dropped below 20 g/L or below 25 g/L for patients suffering from MN.

Patients were treated with a classical regimen of apixaban 5 mg twice daily. In patients fulfilling at least two of the following criteria, a reduced dose of apixaban 2.5 mg twice daily was used: age ≥80 years, body weight ≤60 kg, or serum creatinine ≥133 micromol/L (1.5 mg/dL). Using liquid chromatography/tandem mass spectrometry, apixaban trough Cmin and Cmax levels (2h after ingestion) were measured.

### Outcome

Baseline characteristics and outcomes were identified from patients' records. Baseline characteristics included demographics, plasma albumin, plasma creatinine, estimated glomerular filtration rate (eGFR) according to Chronic Kidney Disease Epidemiology Collaboration (CKD-Epi), low-density lipoprotein (LDL) cholesterol, hemoglobin level, anti-Xa apixaban level, urine protein excretion, and type of glomerular disease. Blood and urine parameters were measured using automated, standardized biological assays. The parameters recorded were measured on the same day as the apixaban level if available. TEs were identified from patients' records and included pulmonary embolism (PE), deep vein thrombosis, renal vein thrombosis, and stroke.

### Statistics

Data were described using mean ± SD (standard deviation) or medians and interquartile range (IQR): 25–75 depending on the distribution.

## Results

The baseline characteristics are shown in Table 1 . A total of 27 consecutive patients were included in the study and 59% were men. Six patients were active smokers, three suffered from diabetes, and eleven had arterial hypertension before NS onset. The mean age at the time of treatment with apixaban was 45.4 years (± 18.3). The mean serum creatinine level was 106.3 (±44.7) µmol/L (normal range between 74.3 and 107 µmol/L). In total, 67% of the patients had an eGFR according to CKD-EPI above 60 mL/min, 11% suffered from chronic kidney disease (CKD) stage 3a, 18% had a CKD stage 3b, and only one patient had CKD stage 4 according to the kidney disease improving global outcome classification criteria. The mean serum albumin was 15.8 g/L (±4.7) (normal range between 35 and 50 g/L), and the median urinary P/C ratio was 7.3 g/g (4.3–10.2) (normal P/C < 0.2 g/g).

**Table 1:** Baseline data

**Number of patients**	27
**Male (%)**	16 (59%)
**Smoking (%)**	6 (22%)
**Diabetes type 2**	3 (11%)
**Arterial hypertension**	11 (41%)
**Mean age in years**	45.4 (± 18.3)
**Mean serum creatinine in µmol/** L	106.3 (± 44.7)
**Mean serum albumin in g/L**	15.8 (4.7)
**Median protein/creatinine ratio in g/g** ( **IQR 25–75** )	7.3 (4.3–10.2)
**Mean LDL cholesterol level in g/** L	2.64 (± 0.8)
**Mean duration of treatment in days**	153 (± 132)
**Number of patients treated with 5 mg** apixaban **twice daily**	20 (74%)
**Mean follow-up time in months since start of treatment with** apixaban	14.7 (± 8.4)
**Renal function (eGFR according to CKD-Epi)**	
**eGFR > 60 m** L **/min**	18 (67%)
**eGFR 45–59 m** L **/min**	3 (11%)
**eGFR 30–44 m** L **/min**	5 (18%)
**eGFR 15–29 m** L **/min**	1 (4%)
**eGFR < 15 m** L **/min**	0
**Patients with venous or arterial TE before start of anticoagulation**	3 (11.1%)
**Patients developing TE during follow-up**	1 (3.7%)
**Use of contraceptive treatment**	0

Abbreviations: CKD-Epi, Chronic Kidney Disease Epidemiology Collaboration; eGFR, estimated glomerular filtration rate; LDL, low-density lipoprotein; TE, thromboembolic events.

Six patients presented minimal change disease, three had the histological diagnosis of focal segmental glomerulosclerosis (FSGS), eleven had anti-PLA2R positive MN, two had anti-PLA2R negative MN, and five suffered from lupus MN. No woman was pregnant or under contraceptive treatment at the time of NS diagnosis.

The mean duration of the anticoagulant treatment was 153 days (±132). Patients were followed for a mean of 14.7 months (±8.4) since the introduction of apixaban. Of note, three patients had a TE at the time of NS diagnosis. Two patients had PE and one patient presented a stroke in lupus MN context. One patient developed PE approximately 2 months after the introduction of apixaban treatment. He suffered from a genetic form of FSGS. Unfortunately, apixaban levels were not measured for this patient at that time. Only one patient was concomitantly treated with aspirin with no bleeding tendency.

**Fig. 1:**
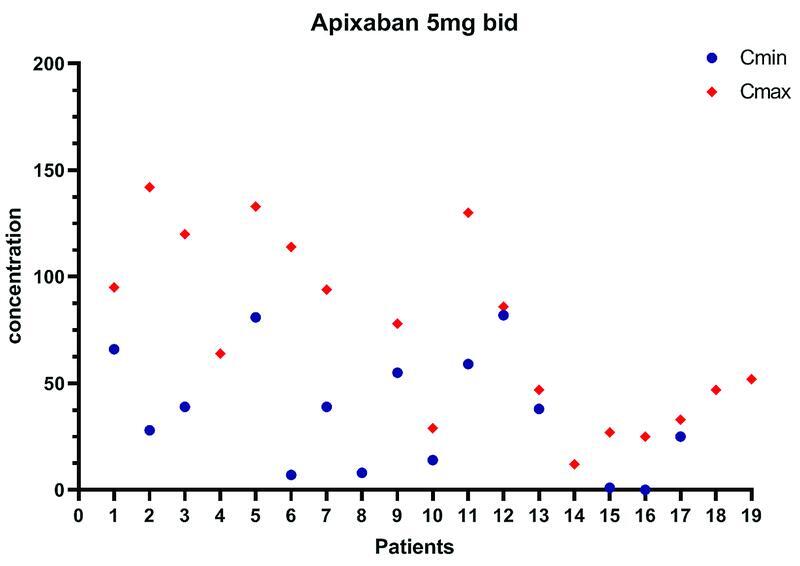
Apixaban trough Cmin and Cmax levels (2 hours after ingestion) are shown for patients on Apixaban 5 mg bid.

**Fig. 2:**
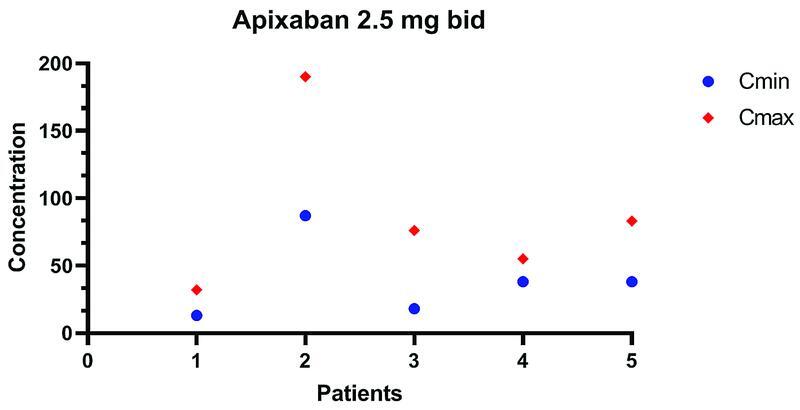
Apixaban trough Cmin and Cmax levels (2 hours after ingestion) are shown for patients on Apixaban 2.5 mg bid.

Figs. 1 and 2 report apixaban Cmin and Cmax determination. Data are missing for three patients. Of note, one patient had only Cmin level measured and 4 patients only Cmax. No minor or major bleeding events were recorded during the follow-up period. Kidney function remained stable during follow-up.

## Discussion

The present study shows that patients suffering from severe NS under anticoagulant therapy with apixaban had a reduced risk of venous and arterial thromboembolism compared with patients previously described in the literature, without increased risk of bleeding.

The DOACs have many advantages over warfarin. In particular, apixaban has a rapid onset (1–3 hours) and offset (half-life 8–15 hours) of action, with predictable dosing, which precludes the need for routine coagulation monitoring in the general population. However, in patients with NS, concerns exist about the optimal dosage of DOACs and their clinical risk/benefit ratio. DOACs are highly protein bound, and hypoalbuminemia could alter blood levels of DOAC with higher free drug levels, precluding possible safety issues. Furthermore, in NS, factor X levels may vary influencing the clinical impact of DOAC 15

Moreover, thromboprophylaxis interest in NS has not been proven in large randomized controlled trials. Evidence is only based on case reports, case series, and retrospective studies using heparin and warfarin as anticoagulants. A recent study by Kelddal et al 5 included 79 patients, of which 44 were treated with low molecular weight heparins and/or warfarin compared with 35 patients without anticoagulation. Four among the thirty-five patients without thromboprophylaxis presented a venous thromboembolic event (VTE) but no patient in the prophylaxis group had thrombosis. 5 Selection bias cannot be excluded since patients without treatment had a higher serum albumin level and no propensity score was used to account for the covariates that could predict receiving the treatment. This study reinforces the clinical interest of thromboprophylaxis in patients with NS. The use of DOACs is tempting and may even obviate the need for concern about heparin resistance due to antithrombin urinary losses. 1

In the current study, we measured for each individual patient Cmin and Cmax anti-Xa apixaban levels. Apixaban was the molecule of choice because of its predictable pharmacokinetic and pharmacodynamic properties that are consistent across the range of different patient populations studied (elderly and those with renal impairment), the fast onset of action, the low the potential for food and drug interactions. 15 We could not compare the levels to previously published studies on apixaban (ARISTOTLE, AMPLIFY, and AMPLIFY-Ext), 16 17 18 since these are the first data gathered in this particular population. However, we notice that the levels are within the therapeutic ranges of the large clinical trials published and observe a normal intraindividual variability of apixaban levels with only one patient experiencing a new TE during follow-up. We must underline the fact that all patients in the study were at high risk of TE events because of the degree of hypoalbuminemia and predominance of MN.

Lionaki et al 19 confirmed that the severity of hypoalbuminemia is significantly associated with VTE occurrence. These authors have shown that each decrease of 10 g/L in albumin serum level is associated with a two-fold increase of VTE risk. A serum albumin level of 28 g/L was the threshold required for anticoagulation implementation. All of the patients included in our study had a serum albumin level < 25 g/L, with only three patients having a serum albumin level above 20 g/L, conferring to this retrospective cohort a significantly high thromboembolic risk.

VTE risk is the highest within the first 6 months after NS onset. The median follow-up time of our study cohort was 11 months (IQR 6–14) and the median duration of anticoagulation was 129 days (IQR: 45–224). Anticoagulation was stopped when serum albumin rose above 20 g/L, with the exception for patients suffering from MN where the threshold was 25 g/L. The follow-up interval was therefore sufficiently large to collect any thrombotic episode

Importantly, no minor or major bleeding adverse events were recorded during follow-up. These findings confirm and extend that anticoagulation with DOACs are safe and well tolerated in patients with severe NS.

Weaknesses of the study were its uncontrolled monocentric historical cohort design, the limited number of patients, and the lack of a control arm comprising heparin and/or warfarin arm.

Although the results of the different studies agree, it must be recognized that the collected evidence to favor the use of DOAC's in NS remains limited because of the small sample size and the retrospective nature of the analysis. However, we retrospectively included all consecutive patients with NS and put on DOACs. This reduces the likelihood of selection bias at the center level. Other weaknesses include a lack of systematic control of the compliance to treatment over the follow-up period and missing data in three patients. Although all patients presented with severe NS, the underlying cause and duration of NS at diagnosis may further confound the results observed. Finally, the introduction of new treatments for the related disease was not systematically investigated.

To the best of our knowledge, this is the first study that measured apixaban levels in patients with NS and where VTE episodes were carefully recorded in the follow-up. We were able to show the encouraging benefit/risk ratio of apixaban in this particular clinical setting in very high-risk patients. Apixaban seems very safe in patients with NS with no bleeding and very effective regarding thrombosis occurrence during a long follow-up. The sole thrombotic episode with PE was reported in a patient with doubtful compliance.

All these data suggest that thromboprophylaxis in patients suffering from severe NS could be simpler and safer and efficacious with apixaban. Further large multicenter randomized, controlled trials should be conducted to confirm these encouraging results.

## Conclusions

The present study shows that patients, suffering from severe NS under anticoagulant therapy with apixaban had a reduced risk of venous and arterial TE compared with patients previously described in the literature, without increased risk of bleeding.

## Notes

**What is Known about the Topic?**

- Few is known about the usage of direct oral anticoagulants in patients with nephrotic syndrome (NS) to prevent thromboembolic events (TEs). Evidence is mostly based on case reports and case series.
- Doubts exist about their efficacy and safety in patients with hypoalbuminemia.

**What does this Paper Add?**

- This is the first study where apixaban levels were monitored in patients with NS caused by glomerular disease.
- The present study shows that patients suffering from severe NS under anticoagulant therapy with apixaban had a reduced risk of venous and arterial TE compared with patients previously described in the literature, without increased risk of bleeding.

## References

[1] Kerlin B A Ayoob R Smoyer W E Epidemiology and pathophysiology of nephrotic syndrome-associated thromboembolic disease Clin J Am Soc Nephrol 2012 7 03 513 520 22344511 10.2215/CJN.10131011 PMC3302669

[2] Barbour S J Greenwald A Djurdjev O Disease-specific risk of venous thromboembolic events is increased in idiopathic glomerulonephritis Kidney Int 2012 81 02 190 195 21918501 10.1038/ki.2011.312

[3] Gyamlani G Molnar M Z Lu J L Sumida K Kalantar-Zadeh K Kovesdy C P Association of serum albumin level and venous thromboembolic events in a large cohort of patients with nephrotic syndrome Nephrol Dial Transplant 2017 32 01 157 164 28391310 10.1093/ndt/gfw227 PMC6251635

[4] Ismail G Mircescu G Ditoiu A V Tacu B D Jurubita R Harza M Risk factors for predicting venous thromboembolism in patients with nephrotic syndrome: focus on haemostasis-related parameters Int Urol Nephrol 2014 46 04 787 792 24078010 10.1007/s11255-013-0574-0

[5] Mahmoodi B K ten Kate M K Waanders F High absolute risks and predictors of venous and arterial thromboembolic events in patients with nephrotic syndrome: results from a large retrospective cohort study Circulation 2008 117 02 224 230 18158362 10.1161/CIRCULATIONAHA.107.716951

[6] Kelddal S Nykjær K M Gregersen J W Birn H Prophylactic anticoagulation in nephrotic syndrome prevents thromboembolic complications BMC Nephrol 2019 20 01 139 31023275 10.1186/s12882-019-1336-8 PMC6482554

[7] Medjeral-Thomas N Ziaj S Condon M Retrospective analysis of a novel regimen for the prevention of venous thromboembolism in nephrotic syndrome Clin J Am Soc Nephrol 2014 9 03 478 483 24334865 10.2215/CJN.07190713 PMC3944768

[8] Rostoker G Durand-Zaleski I Petit-Phar M Prevention of thrombotic complications of the nephrotic syndrome by the low-molecular-weight heparin enoxaparin Nephron J 1995 69 01 20 28 10.1159/000188355 7891793

[9] Sexton D J de Freitas D G Little M A Direct-acting oral anticoagulants as prophylaxis against thromboembolism in the nephrotic syndrome Kidney Int Rep 2018 3 04 784 793 29989039 10.1016/j.ekir.2018.02.010 PMC6035159

[10] Han T HC Thet Z Warfarin *vs* . new oral anticoagulant in primary adult nephrotic syndrome associated venous thromboembolism Nephrology (Carlton) 2017 22 64

[11] Matta A Elenizi K AlHarthi R Moussallem N Elhajjaji N Lhermusier T Carrié D A Case of Isolated Unilateral Right Renal Vein Thrombosis Associated with Bilateral Pulmonary Embolism Treated with Rivaroxaban a Direct-Acting Oral AnticoagulantAm J Case Rep 2019 Aug 6; 20:1152–1154. Doi 10.12659/AJCR.916638 PMID: 31383838; PMCID: PMC6691838 10.12659/AJCR.916638 PMC6691838 31383838

[12] Zhang L Zhang H Zhang J Tian H Liang J Liu Z Rivaroxaban for the treatment of venous thromboembolism in patients with nephrotic syndrome and low AT-III: a pilot study Exp Ther Med 2018 15 01 739 744 29399079 10.3892/etm.2017.5471 PMC5772665

[13] Dupree L H Reddy P Use of rivaroxaban in a patient with history of nephrotic syndrome and hypercoagulability Ann Pharmacother 2014 48 12 1655 1658 25169250 10.1177/1060028014549349

[14] Ramos-Esquivel A Monitoring anticoagulant therapy with new oral agents World J Methodol 2015 5 04 212 215 26713281 10.5662/wjm.v5.i4.212 PMC4686418

[15] Byon W Garonzik S Boyd R A Frost C E Apixaban: a clinical pharmacokinetic and pharmacodynamic review Clin Pharmacokinet 2019 58 10 1265 1279 31089975 10.1007/s40262-019-00775-z PMC6769096

[16] ARISTOTLE Committees and Investigators Granger C B Alexander J H McMurray J JV Apixaban versus warfarin in patients with atrial fibrillation N Engl J Med 2011 365 11 981 992 21870978 10.1056/NEJMoa1107039

[17] AMPLIFY Investigators Agnelli G Buller H R Cohen A Oral apixaban for the treatment of acute venous thromboembolism N Engl J Med 2013 369 09 799 808 23808982 10.1056/NEJMoa1302507

[18] AMPLIFY-EXT Investigators Agnelli G Buller H R Cohen A Apixaban for extended treatment of venous thromboembolism N Engl J Med 2013 368 08 699 708 23216615 10.1056/NEJMoa1207541

[19] Lionaki S Derebail V K Hogan S L Venous thromboembolism in patients with membranous nephropathy Clin J Am Soc Nephrol 2012 7 01 43 51 22076873 10.2215/CJN.04250511 PMC3265338

